# Validity and reliability of the single camera marker less motion capture system using RGB-D sensor to measure shoulder range-of-motion: A protocol for systematic review and meta-analysis

**DOI:** 10.1097/MD.0000000000033893

**Published:** 2023-06-02

**Authors:** Unhyung Lee, Suji Lee, Sung-A Kim, Jae-Dong Lee, Seunghoon Lee

**Affiliations:** a Department of Clinical Korean Medicine, Graduate School, Kyung Hee University, Seoul, Republic of Korea; b Department of Acupuncture and Moxibustion Medicine, Kyung Hee University Medical Center, Seoul, Republic of Korea; c Department of Acupuncture and Moxibustion, College of Korean Medicine, Kyung Hee University, Seoul, Republic of Korea.

**Keywords:** protocol, range-of motion, RGB-D sensor, shoulder, systematic review

## Abstract

The range-of motion (ROM) is an essential component of joint mobility. Shoulder ROM measurement has been problematic due to its complexity. A marker less motion capture system can be a potential alternative for upper limb assessment. Currently, there is no systematic review to evaluate the validity of a marker less motion capture system for assessing shoulder ROM. This study aims to describe methods to evaluate the reliability and validity of a single camera marker less motion capture system that uses an RGB-depth sensor to measure shoulder ROM.

**Methods:**

Studies that measured shoulder ROM with a single camera marker less motion capture system using the RGB-depth sensor and assessed the intra- and/or inter-rater reliability, and/or validity of the device will be included. The search of electronic databases, such as MEDLINE, EMBASE, Cochran library, Cumulative Index to Nursing, and Allied Health Literature via EBSCO, IEEE Xplore, China National Knowledge Infrastructure, KoreaMed, Korean studies Information Service System, and Research Information Sharing Services will be performed for all relevant articles from inception to December 2022. Two authors will independently perform quality assessments using the Consensus-based Standards for the selection of health Measurement Instruments checklist for reliability, measurement error of outcome measurement instrument, and criterion validity. The primary outcomes will be the intra- and inter-rater reliability and validity of the markerless motion capture system measuring shoulder flexion, extension, abduction, adduction, internal rotation, or external rotation. A subgroup analysis would be performed if there are sufficient data to pool to identify an influencing factor in the measurement of ROM using a marker less motion capture system.

**Results and Conclusion:**

These findings will present tools to utilize and evaluate single camera motion capture systems for the medical use for clinicians and healthcare experts and can aid in further clinical research using such a system for different movements and other joints.

## 1. Introduction

The range-of motion (ROM) is an essential component of joint mobility. It indicates the current pathologic or physiologic state of the joint and effect of treatment by comparing ROM before and after treatment. Specifically, shoulder motion can be a differentiator for various shoulder disorders, including rotator cuff tear, adhesive capsulitis, and impingement syndrome.^[1]^ Therefore, measuring shoulder ROM is important in clinical practice.

The shoulder joint complex consists of the acromioclavicular, glenohumeral, scapulothoracic, and sternoclavicular joints, and the motion is made up of the coordination of all these joints. Because of this complexity, measuring shoulder motion is complex and difficult to apply consistently.^[2,3]^ Therefore, an accurate and consistent measurement method is needed.

Conventionally, goniometry is the most commonly used method for measuring joint ROM. However, its low inter-rater reliability and measurement variability between clinicians make it difficult to apply in clinical settings.^[4]^ Although a 3D-marker based motion tracking system is used as an alternative, it is expensive, requires a large space, and requires time and clinician’s experience. These drawbacks make traditional motion capture systems unsuitable for clinical use.^[5]^

In comparison, a marker less motion capture system can be possible alternative for upper limb assessment. The marker less motion capture system is a low-cost motion capture system with an RGB camera and a depth camera. The sensor was initially commercialized as an add-on (Microsoft Kinect) to the X-box 360 console (Microsoft Corp., Redmond, WA) and modified for various fields including kinematic motion analysis.^[6]^ The sensor can detect joint orientation and track joints and skeletal positions, and this makes it possible to analyze joint motion and kinematics without space and monetary limitations.^[4]^ This system can hence be a possible alternative to goniometry and traditional motion capture systems for upper limb assessment.

For ROM assessment devices, the reliability and validity of measurement are key characteristics for clinical use.^[7]^ Several studies^[8–10]^ have reported the validity and reliability of marker less motion capture systems in measuring ROM, but the measurement characteristics vary and the results are inconsistent. There is only 1 systematic review on shoulder ROM using a marker less motion capture system, but it compared only reliability with the intraclass correlation coefficient (ICC)^[4]^; there is no systematic review on assessment of validity of marker less motion analysis system for shoulder ROM. Therefore, we aim to perform a systematic review of studies that measured reliability and validity of a single camera marker less motion capture system using an RGB-depth (RGB-D) sensor to measure shoulder ROM.

## 2. Methods

### 2.1. Study registration

This systematic review and meta-analysis protocol is reported in accordance with the preferred reporting items for systematic reviews and meta-analysis protocol guidelines.^[11]^ The protocol of the review was registered online at PROSPERO (CRD42023395441) on February 2022.

### 2.2. Inclusion and exclusion criteria for study selection

Studies that measured shoulder ROM using a single camera marker less motion capture system with an RGB-D sensor and assessed the intra- and/or inter-rater reliability, and/or validity of the device will be included. Among the studies that measured ROM using Microsoft Kinect, those that evaluated Microsoft Kinect V1 will be excluded in this review because Kinect V1 measures depth using the pattern projection principle, whereas Kinect V2 and Azure Kinect measure depth using the continuous wave intensity modulation approach, which is most commonly used in time-of-flight cameras.^[12]^ In addition, several studies have found Kinect V2 to be more valid and reliable for measuring ROM.^[5,13]^ Studies on healthy subjects and patients with shoulder disorders or upper limb motor disorder in all ages will be included.

Case studies, reviews, studies without full text, or studies that do not measure shoulder joints by angle will be excluded.

### 2.3. Types of outcome measures

The main outcomes of this study will be the intra- and inter-rater reliability and validity of the marker less motion capture system measuring shoulder flexion, extension, abduction, adduction, internal rotation, or external rotation. If there are active and passive ROM values, these will be considered the main outcomes.

### 2.4. Search strategy

A database search of MEDLINE, EMBASE, Cochran library, Cumulative Index to Nursing and Allied Health Literature via EBSCO, IEEE Xplore, China National Knowledge Infrastructure, KoreaMed, Korean studies Information Service System, and Research Information Sharing Services will be performed by 2 independent reviewers. The search strategy consists of 3 parts: RGB-D sensor (e.g., Kinect, RGB-D camera, or infrared), shoulder (e.g., shoulder, upper limb, upper, or extremity), and ROM (e.g., range-of motion, ROM, or kinematics). Details of the search strategy are presented in Supplemental Digital Content, http://links.lww.com/MD/J37. These databases will be searched from inception to December 2022.

If a study meeting the inclusion criteria is found outside the search area, it will be additionally included with the 2 reviewers consent.

### 2.5. Data collection and analysis

#### 2.5.1. Selection of studies

The full texts will be retrieved using the search strategy. Two review authors (UL and SL) will read the title and abstract and screen for eligibility using the predefined criteria. Any disagreements on eligibility will be resolved through discussion with involvement of a third review author (SL) if necessary. Full text versions that meet the criteria will be uploaded Endnote20.

#### 2.5.2. Data extraction and analysis

Two independent reviewers (UL and SL) will extract data, including basic study information (author names and year of publication), information on population characteristics (mean age of participants, sex, height, mass of participants, and sample size), device (type and description of the camera and software), and study design (measurement method of a comparison, required movement, position of the subject, results of intra- and inter-rater reliability, results of validity, statistical method, number of raters, number of sessions, and session interval).

#### 2.5.3. Quality assessment

For the quality assessment of reliability and validity studies, 2 reviewers (UL and SL) will independently assess 3 measurement properties from the consensus-based standards for the selection of health measurement instruments (COSMIN) checklist: reliability, measurement error of outcome measurement instrument, and criterion validity.^[14]^ As COSMIN is originally used as a patient-reported outcome measure, the extended version of COSMIN is recommended for reliability and measurement error. Therefore, in this study, 9 standards for reliability, 8 standards for measurement error, and 3 standards for validity will be assessed by 4-point scale, and the method with the worst score will be applied for grading. Any disagreement on eligibility will be resolved through discussion with the involvement of a third review author (SL) if necessary.

#### 2.5.4. Data synthesis

The principal analysis will be narratively assessed with the intra- or inter-rater reliability and validity for a single camera marker less motion capture system using an RGB-D sensor to measure the joint angle of the shoulder. All results will be summarized as a table. The heterogeneity will be identified by the I2 statistics to be evaluated to quantify the inconsistency among the included studies. These values will be interpreted as follows: unimportant heterogeneity (0%–40%), moderate heterogeneity (30%–60%), substantial heterogeneity (50%–90%) and considerable heterogeneity (75%–100%).^[15]^ If significant heterogeneity is identified, only descriptive synthesis will be performed without performing quantitative synthesis.

The R program (version 4.2.1)^[16]^ will be used to perform meta-analysis. The pooled effect estimate will be calculated as a weighted average and a random effects model will be used to deal with potential heterogeneity among included studies. The correlation coefficients will be converted to Fisher z scores and then pooled. Fisher z scores will be then converted back into the ICC values. Forest plots with 95% confidence intervals will be generated to summarize the results. The data are expected to be pooled using the ICC values for intra- and inter-rater reliability, and using the Pearson correlation coefficients for validity. Additionally, correlation values are interpreted as follow: a coefficient <0.5 indicated poor, from 0.5 to 0.75 indicated moderate, from 0.75 to 0.9 indicated good, and values more than 0.90 indicated excellent.^[17]^

#### 2.5.5. Subgroup analysis

Subgroup analysis is planned according to the following if there are sufficient data that can be pooled to identify the influencing factor of measuring ROM using a marker less motion capture system. Subgroup analysis will focus on details of the study design that may affect the reliability and validity based on study size [e.g. relatively large study (participants ≥ 30) and small study (participants < 30)], type of camera (e.g., Kinect V2, Azure Kinect, or Intel Realsense), type of movement (e.g. active ROM, passive ROM, or both), direction of movement (flexion, extension, abduction, adduction, external rotation, and internal rotation), subject’s position (e.g., sitting/standing, and frontal/lateral), and subject’s health status affecting shoulder function (e.g., patients with shoulder disorder and healthy subjects).

#### 2.5.6. Assessment of reporting biases

The funnel plot will be used to assess reporting bias when more than 10 trials are included.^[15]^

### 2.6. Ethics and dissemination

Since we will conduct a systematic review, consent and ethical approval will not be needed. The review will be distributed through conference presentations or peer-reviewed journals.

## 3. Discussion

The RGB-D camera used in single camera marker less motion capture system is technically improving and has been utilized in various industries such as fitness,^[18]^ sports,^[19]^ and digital therapeutics.^[20]^ However, for medical utilization, high reliability and validity should be ensured because repeated assessments are used to determine the function of the joint at baseline, during treatment, and after treatment.^[21]^ Since the shoulder joint is complex and has the largest ROM among joints of the human body,^[22]^ the reliability and validity of shoulder ROM measurement have been a concern. Therefore, this study aims to systematically review studies that have measured reliability and validity of a single camera motion capture system assessing shoulder ROM and investigate factors that may affect the results.

Azure Kinect has less noise and better accuracy than Kinect V2.^[12]^ Clinically, Albert et al^[23]^ reported significantly higher pose tracking accuracy using Azure Kinect compared to that using Kinect V2. In such studies, the type of device may affect the reliability and validity of the measurement; thus, the present study will analyze results by device in subgroup analysis. Lee et al^[24]^ reported that sitting and standing positions may cause differences in shoulder ROM measurements, and Beshara et al^[4]^ emphasized appropriate patient posture and protocol standardization for measuring shoulder ROM. For these reasons, this study aims to analyze measurement methods that may affect validity and reliability, including patient and sensor location in the subgroup analysis.

Through this proposed study, the authors hope to provide clinicians and healthcare experts with tools to utilize and evaluate single camera motion capture systems for the medical use. In addition, this study will provide guidance for a further clinical study using a single camera motion capture system for different movements and other joints.

## Acknowledgments

We would like to thank Seongsu Joo and EunSik Park for their suggestions on technical aspects of single camera motion capture systems.

## Author contributions

**Conceptualization:** Jae-Dong Lee, Seunghoon Lee.

**Methodology:** Sung-A Kim.

**Writing – original draft:** Unhyung Lee, Suji Lee, Seunghoon Lee.

**Writing – review & editing:** Seunghoon Lee.

## Supplementary Material


