# Supplementary material for: Validity and reliability of the single camera marker less motion capture system using RGB-D sensor to measure shoulder range-of-motion: A protocol for systematic review and meta-analysis
Source: Medicine (Baltimore). 2023 Jun 2;102(22):e33893. doi: 10.1097/MD.0000000000033893 (PMC10238018; doi:10.1097/MD.0000000000033893)
Supplement: Supplementary file 1 [file medi-102-e33893-s001.pdf]

Supplemental Digital Content _ Search strategy

1. MEDLINE via PubMed

#1 Search all field for “kinect” OR “microsoft kinect” OR “infrared” OR “RGB-D camera” OR “RGB-D sensor” OR “RGB-D” OR “RGB-depth” OR “depth camera” OR “3D camera”

#2 Search all field for “shoulder” OR “upper limb” OR “upper body” OR “upper extremity”

#3 Search all field for “range of motion” OR “kinematic” OR “motor” OR “movement” OR “angle” OR “motion”

#4 #1 AND #2 AND #3

2. EMBASE

#1 ‘motion analysis system’/exp OR ‘motion analysis system’:ab,ti

#2 infrared:ab,ti

#3 ‘RGB-D’:ab,ti

#4 ‘RGB-Depth’:ab,ti

#5 ‘depth camera’:ab,ti

#6 ‘3D camera’:ab,ti

#7 #1 OR #2 OR #3 OR #4 OR #5 OR #6

#8 ‘shoulder’:ab,ti

#9 ‘upper limb’:ab,ti

#10 ‘upper body’:ab,ti

#11 ‘upper extremity’:ab,ti

#12 #8 OR #9 OR #10 OR #11

#13 ‘range of motion’/exp OR ‘range of motion’:ab,ti

#14 kinematic:ab,ti

#15 motor:ab,ti

#16 movement:ab,ti

#17 angle:ab,ti

#18 motion:ab,ti

#19 #13 OR #14 OR #15 OR #16 OR #17 OR #18

#20 #7 AND #12 AND #19

3. Cochrane library

#1 Search all field for “kinect” OR “microsoft kinect” OR “infrared” OR “RGB-D camera” OR “RGB-D sensor” OR “RGB-D” OR “RGB-depth” OR “depth camera” OR “3D camera”

#2 Search all field for “shoulder” OR “upper limb” OR “upper body” OR “upper extremity”

#3 Search all field for “range of motion” OR “kinematic” OR “motor” OR “movement” OR “angle” OR

“motion”

#4 Search MeSH term “range of motion, articular”

#5 #3 OR #4

#6 #1 AND #2 AND #5
